# TiO_2_/GO-coated functional separator to suppress polysulfide migration in lithium–sulfur batteries

**DOI:** 10.3762/bjnano.10.168

**Published:** 2019-08-19

**Authors:** Ning Liu, Lu Wang, Taizhe Tan, Yan Zhao, Yongguang Zhang

**Affiliations:** 1 School of Materials Science and Engineering, Hebei University of Technology, Tianjin 300130, China; 2 Synergy Innovation Institute of GDUT, Heyuan 517000, China

**Keywords:** dealloying, functional separator, lithium–sulfur batteries, TiO_2_/GO composite

## Abstract

Lithium–sulfur batteries render a high energy density but suffer from poor cyclic performance due to the dissolution of intermediate polysulfides. Herein, a lightweight nanoporous TiO_2_ and graphene oxide (GO) composite is prepared and utilized as an interlayer between a Li anode and a sulfur cathode to suppress the polysulfide migration and improve the electrochemical performance of Li/S batteries. The interlayer can capture the polysulfides due to the presence of oxygen functional groups and formation of chemical bonds. The hierarchically porous TiO_2_ nanoparticles are tightly wrapped in GO sheets and facilitate the polysulfide storage and chemical absorption. The excellent adhesion between TiO_2_ nanoparticles and GO sheets resulted in enhanced conductivity, which is highly desirable for an efficient electron transfer process. The Li/S battery with a TiO_2_/GO-coated separator exhibited a high initial discharge capacity of 1102.8 mAh g^−1^ and a 100th cycle capacity of 843.4 mAh g^−1^, which corresponds to a capacity retention of 76.48% at a current rate of 0.2 C. Moreover, the Li/S battery with the TiO_2_/GO-coated separator showed superior cyclic performance and excellent rate capability, which shows the promise of the TiO_2_/GO composite in next-generation Li/S batteries.

## Introduction

The portability of handheld electronic products and successful realization of next-generation electric vehicles urgently require advanced energy storage devices with higher storage capacity and excellent service life. Li-ion batteries have successfully demonstrated their promise for a wide range of small-scale applications. However, the large-scale utilization of Li-ion batteries is limited by the energy density [1–5]. Recently, lithium–sulfur batteries (Li/S batteries) have been widely investigated as an alternative energy storage system due to their distinct advantages, such as high theoretical capacity (1675 mAh g^−1^) and high energy density (2600 Wh kg^−1^). Furthermore, the abundance and nontoxic nature of elemental sulfur favors the large-scale utilization of Li/S batteries [6–10]. However, the development and widespread utilization of Li/S batteries is hindered by (i) the poor electronic/ionic conductivity of sulfur, causing a low reaction rate and electrochemical polarization, (ii) dissolution and the shuttle effect of intermediate polysulfides, resulting in the deposition of Li_2_S and Li_2_S_2_ at the electrode/electrolyte interface, shortening the service life and rendering poor coulombic efficiency, and (iii) large volumetric changes during charge/discharge, destroying the conductive network of the electrode and causing capacity decay [11–15].

To overcome these issues, researchers have adopted various techniques, such as optimization of the cathode material [16–18], incorporation of electrolyte additives [19], and protection of the anode [20]. Recently, much attention has been directed to the development of a functional separator, which serves as an intermediate layer and plays an important role in enhancing the electrochemical performance of Li/S batteries. It has been demonstrated that the polysulfide shuttle can be effectively suppressed by modifying the separator or incorporating an interlayer at the cathode/separator interface [21–22]. For instance, the performance of Li/S batteries has been significantly enhanced by using carbon-modified separators due to the superior conductivity, adjustable pore structure and high specific surface area [23–26]. However, only physical adsorption occurs between carbonaceous materials and polysulfides, and nonpolar carbon-based materials offer weak interactions with polar polysulfides [27–28]. On the other hand, metal oxides can form chemical bonds with sulfur to trap sulfur species. As a result, sulfur species are confined at the cathode/separator interface and the shuttle effect is minimized. Hence, the carbon/metal-oxide hybrid interlayer combines the advantages of carbon and metal oxides and exhibits superior performance over monolithic materials. Recently, the inclusion of V_2_O_5_/CNT [29], MoO_3_@CNT [30], TiO_2_@CNF [31], TiO_2_/graphene [32] interlayers has been shown to suppress the shuttle effect and the Li/S batteries with these functional interlayers deliver high gravimetric energy density and superior cyclic performance.

Two-dimensional graphene oxide (GO) has excellent thermal stability, an ultrahigh specific surface area, and good electrical conductivity. The polysulfide shuttle can be suppressed due to the presence of oxygen functional groups on the surface of GO, electrostatic repulsion and steric exclusion [33–34]. However, as far as we know, no study has been reported applying TiO_2_/GO composites as a functional interlayer in Li/S batteries. Herein, a three-dimensional TiO_2_/GO-coated separator was introduced between the Li anode and sulfur cathode as a highly efficient polysulfide absorber. The TiO_2_/GO composite was prepared by dealloying, as reported elsewhere [35], and subsequent spray drying. It has been demonstrated that the utilization of the TiO_2_/GO composite interlayer enhanced the cycling stability and charge storage capacity of Li/S batteries due to excellent conductivity of graphene oxide and strong chemical interactions between nanoporous TiO_2_ and polysulfides.

## Results and Discussion

**Figure 1:**
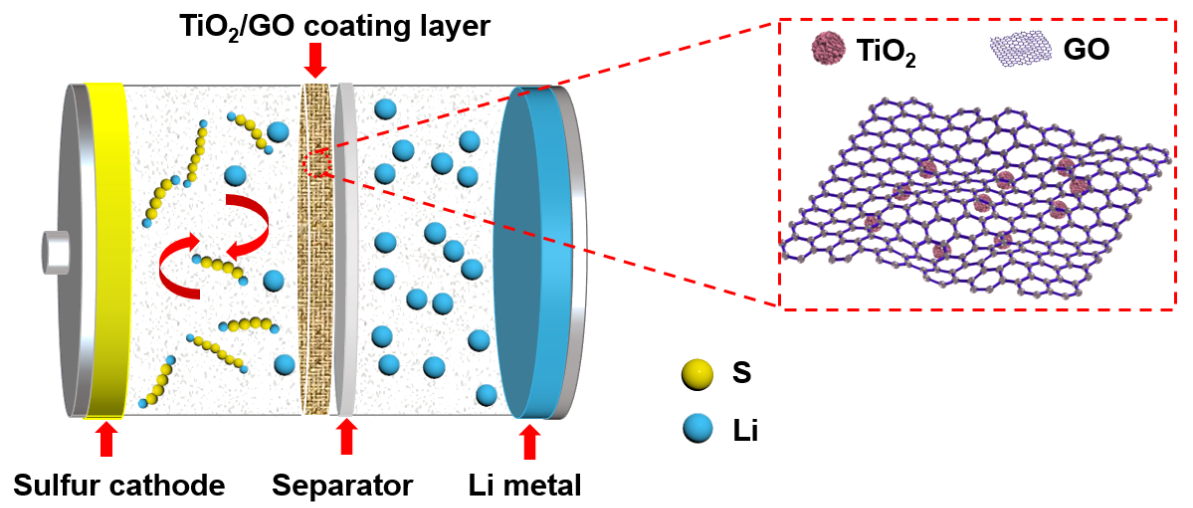
Schematic illustration of a Li/S battery with a TiO_2_/GO-coated functional separator.

Figure 1 presents a schematic of a Li/S battery with a TiO_2_/GO-coated separator, which is sandwiched between a sulfur cathode and Li metal and prevents the diffusion of polysulfides. Thereby, the separator inhibits the polysulfide shuttle during the charge/discharge process. At the same time, the coating layer provides an unimpeded pathway for the transmission of Li ions, which guarantees the excellent cyclic stability and desirable rate performance of Li/S batteries.

**Figure 2:**
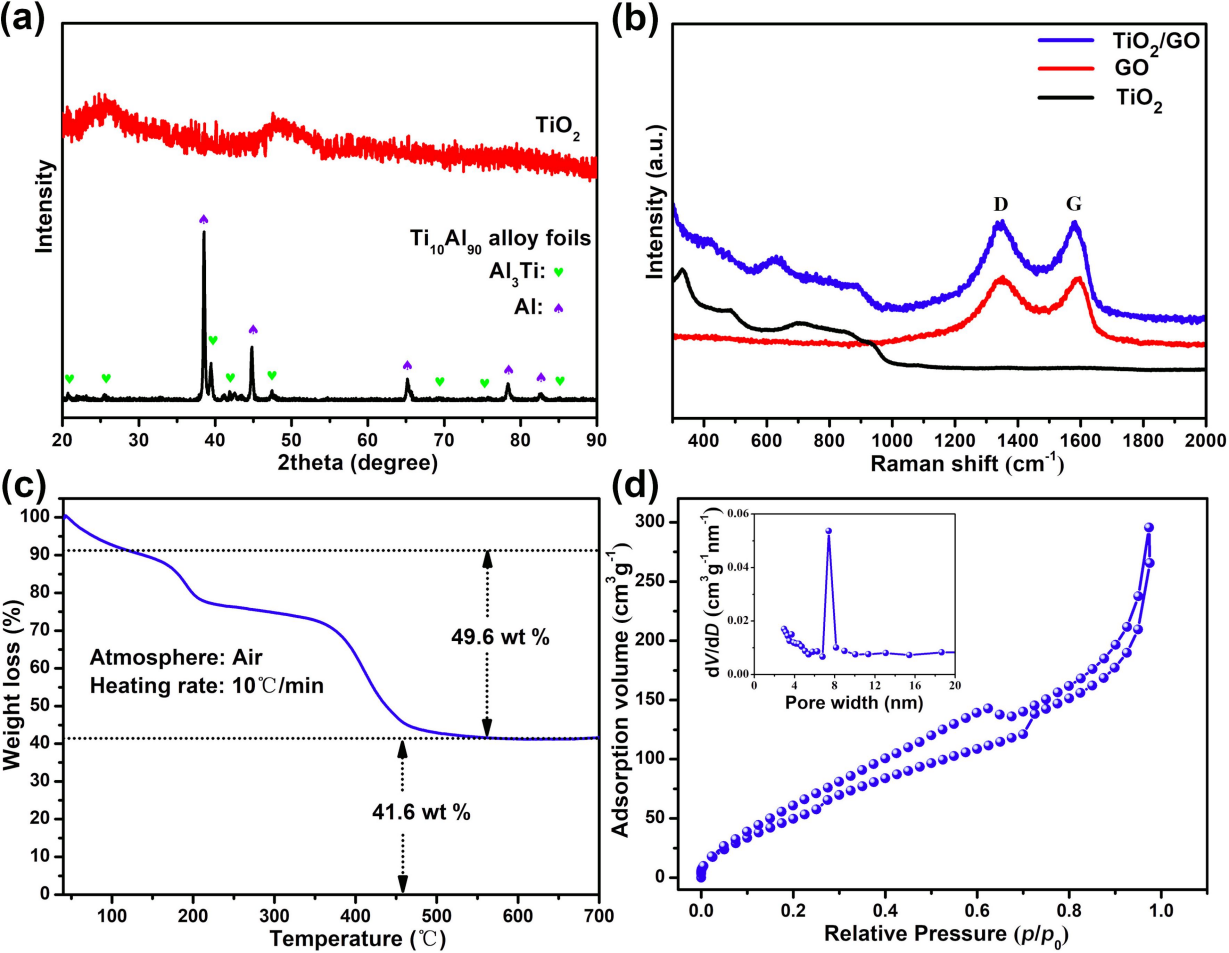
(a) XRD patterns of the as-spun and as-dealloyed Ti_10_Al_90_ alloy foils. (b) Raman spectra of TiO_2_, GO and the TiO_2_/GO composite. (c) TGA curve and (d) N_2_ adsorption–desorption isotherms and pore size distribution of the TiO_2_/GO composite.

Figure 2a shows X-ray diffraction (XRD) patterns of the as-spun and as-dealloyed sample. The TiAl foil exhibits Al_3_Ti (JCPDS 65-2667) and Al (JCPDS 65-2869) phases. After dealloying, the specimen shows a typical amorphous state with two weak diffraction peaks at about 25° and 48°, and the peaks of the Al_3_Ti and Al phases were absent, indicating almost complete dissolution of Al and the formation of amorphous TiO_2_. Figure 2b shows the Raman spectra of TiO_2_, GO and the TiO_2_/GO composite. The Raman spectrum of as-dealloyed TiO_2_ is featureless due to its amorphous nature [36], and the GO alone shows the typical D- and G-bands at ≈1350 cm^−1^ and 1592 cm^−1^. Meanwhile, the composite displays the spectral characteristics of GO with two distinct peaks at ≈1343 cm^−1^ and 1580 cm^−1^. The slight shift in the position of the D- and G-band of the TiO_2_/GO composite can be ascribed to the interaction between TiO_2_ and GO and the formation of Ti–O–C bonds [37]. In addition, the TiO_2_/GO composite shows a new, weak peak at 628 cm^−1^ that corresponds to the *E*_g_ mode of the anatase TiO_2_ [38], suggesting that the TiO_2_ is crystallized with a low degree of crystallinity after spray drying. Thermogravimetric analysis (TGA) of the TiO_2_/GO composite is presented in Figure 2c. When the temperature was increased from room temperature to 120 °C, a weight decrease of 8.8% was noticed due to the elimination of a small amount of adsorbed water. The remainder after the heating process was regarded as the TiO_2_, which accounts for 45.6 wt % of the whole. The N_2_ adsorption–desorption isotherm of the TiO_2_/GO composite is shown in Figure 2d. A distinct hysteresis loop can be identified, indicating the microporous structure of the TiO_2_/GO composite. The BET specific surface area of the TiO_2_/GO composite was determined to be 155.2 m^2^ g^−1^. Through the Barrett–Joyner–Halenda (BJH) analysis, the pore size distribution of TiO_2_/GO shows that the majority of the pores are around 2.9 and 7.4 nm. The rich porosity not only provides abundant pore structure to accommodate sulfur, but also supplies numerous adsorption and catalytic sites for the polysulfides, thus significantly improving both the specific capacity and cycling performance of Li/S batteries.

**Figure 3:**
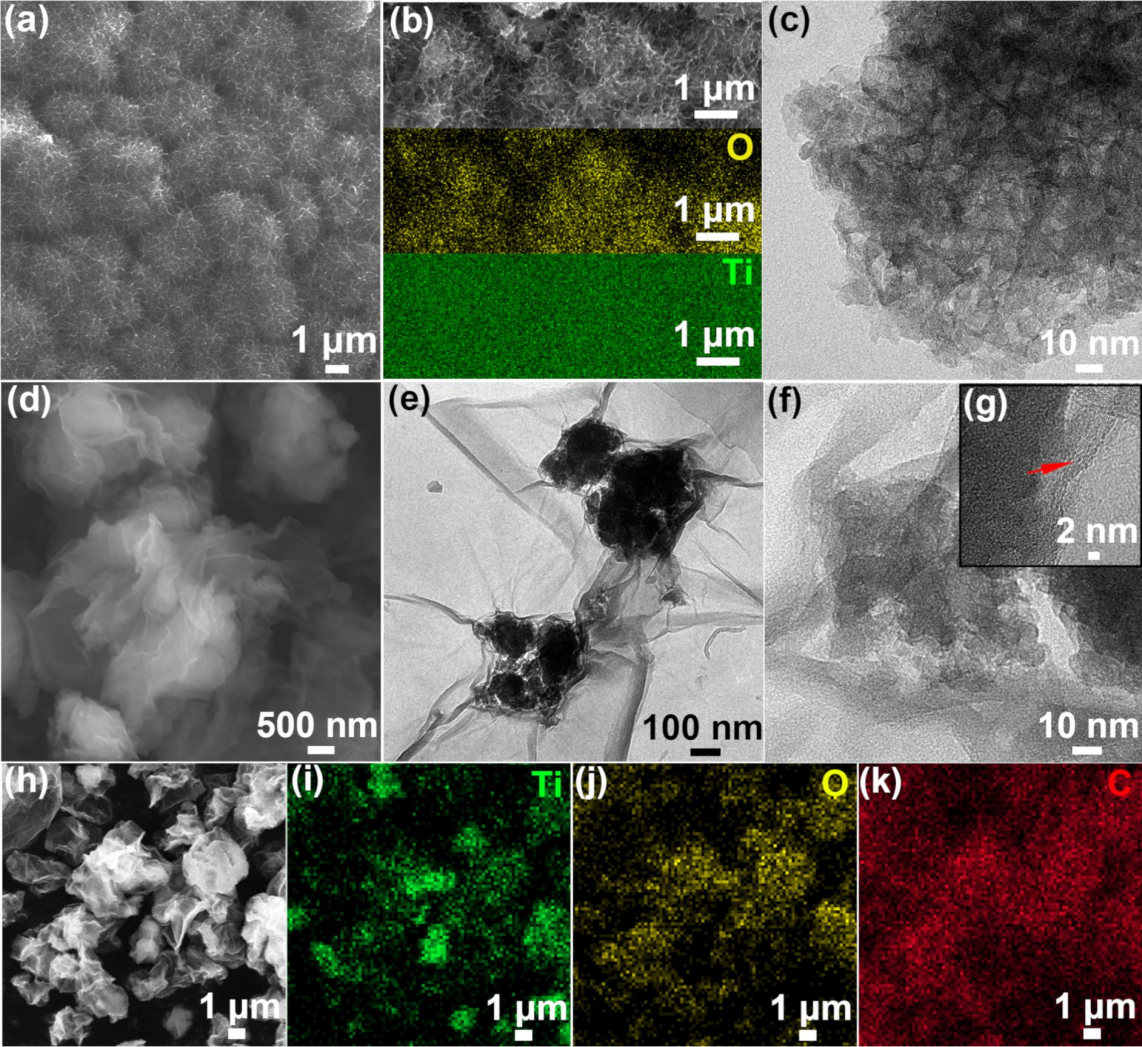
(a) SEM image, (b) element maps and (c) TEM image of the as-prepared nanoporous TiO_2_ particles. (d) SEM image, (e) TEM image, (f, g) HRTEM images and (h–k) EDS mapping of the as-prepared TiO_2_/GO composite.

Figure 3a shows a scanning electron microscopy (SEM) image of as-prepared TiO_2_, which has been synthesized by selectively dissolving Al atoms from a Ti_10_Al_90_ alloy. It can be readily observed that the abundant nanowires and uniform nanopores, with a pore size of ≈40 nm, formed a sea-urchin-like structure. Furthermore, the energy-dispersive X-ray spectroscopy (EDS) analysis confirms the homogenous distribution of Ti and O elements (Figure 3b). In addition, the transmission electron microscopy (TEM) image of TiO_2_ shows the nanoporous architecture with dark nanowires and bright nanopores (Figure 3c). On the other hand, the SEM and TEM images of the as-prepared TiO_2_/GO composite show that the surface of nanoporous TiO_2_ has been completely wrapped by wrinkled GO nanosheets (Figure 3d and 3e). As displayed in the high-resolution TEM (HRTEM) images (Figure 3f and 3g), the TiO_2_/GO composite reveals no clear lattice fringe for TiO_2_, indicating poor crystallinity. It is clear that the GO sheets have a flake-like structure with wrinkles and folds, which is in line with previous works [39]. The EDS elemental mapping of titanium, oxygen and carbon provide additional evidence to further show the GO uniform distribution on the TiO_2_ particle, as shown in Figure 3i–k. Moreover, based on the Raman and TEM results, the TiO_2_ and GO sheets exhibit excellent adhesion, which ensures efficient electron transfer from the GO sheet to nanoporous TiO_2_. The use of TiO_2_/GO composites as an interlayer can greatly suppress the migration of polysulfides due to their physical and chemical interactions with dissolved polysulfides. Therefore, the as-prepared TiO_2_/GO composite is expected to exhibit enhanced conductivity and render excellent rate performance [40].

**Figure 4:**
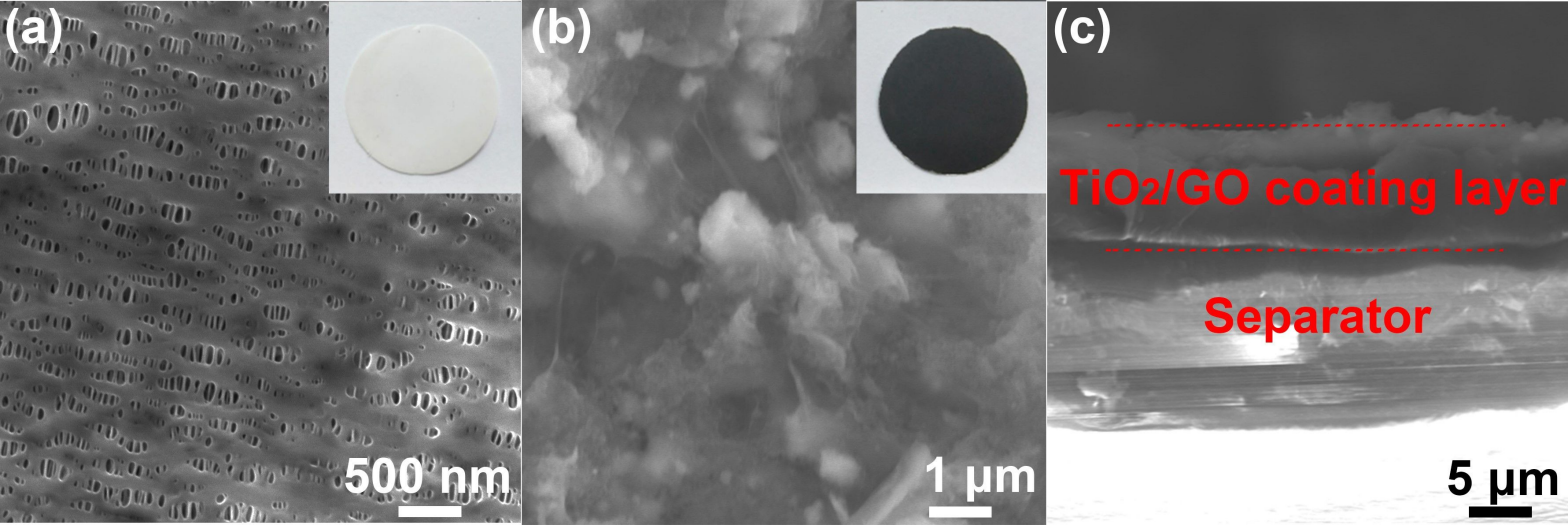
Surface SEM images of (a) a pristine separator, (b) a TiO_2_/GO-coated separator and (c) the TiO_2_/GO-coated separator as a cross-sectional view. Insets in panel (a) and (b) are the digital photographs of the pristine and the TiO_2_/GO-coated separator, respectively.

Figure 4 displays the SEM images of the pristine and TiO_2_/GO-coated separator. The pristine separator shows abundant pores with an average diameter of 100 nm (Figure 4a). Meanwhile, the TiO_2_/GO-coated separator confirms that TiO_2_ particles are tightly wrapped with GO sheets, indicating the strong interaction between nanoporous TiO_2_ and GO (Figure 4b). The cross-sectional morphology of the TiO_2_/GO-coated separator shows that the thickness of the TiO_2_/GO composite layer was ≈5 μm (Figure 4c). Furthermore, the interface does not contain any cracks, suggesting the excellent adhesion of the TiO_2_/GO composite layer with a pristine separator.

**Figure 5:**
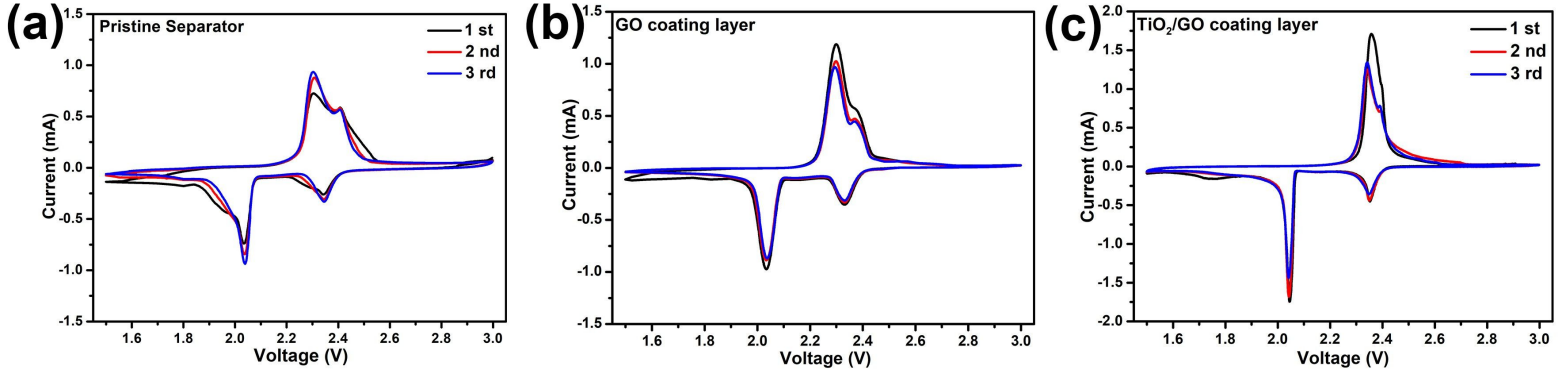
CV curves of the Li/S batteries with a (a) pristine separator, (b) GO-coated separator and (c) TiO_2_/GO-coated separator.

Figure 5 compares the cycle voltammetry (CV) curves of the Li/S batteries with pristine, GO-coated and TiO_2_/GO-coated separators at a scan rate of 0.1 mV s^−1^. All the CV curves exhibit two cathodic peaks, which can be ascribed to the transformation of elemental sulfur, S_8_, into soluble high-order polysulfides and then into Li_2_S and Li_2_S_2_ [41]. On the other hand, the anodic peaks can be assigned to the reversible transformation of Li_2_S and Li_2_S_2_ into the high valence state Li_2_S_4–8_ [42–44]. One should note that the Li/S batteries with a pristine separator and those with a GO-coated separator exhibit much broader redox peaks than that of the Li/S batteries with TiO_2_/GO-coated separator due to the high polarization and poor reversibility. Moreover, compared with Figure 5a, the position and shape of redox for the 2nd and 3rd cycles remain unchanged in Figure 5c. In addition, the batteries with the TiO_2_/GO-coated separator exhibit sharper and more symmetric redox peaks than the batteries with a pristine separator or a GO-coated separator. These observations indicate the stable electrochemical performance and high reversibility of the Li/S batteries with the TiO_2_/GO-coated separator.

**Figure 6:**
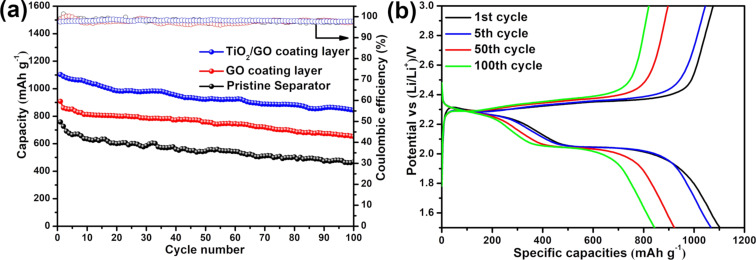
(a) Cyclic performance and coulombic efficiency of the Li/S batteries with pristine, GO-coated and TiO_2_/GO-coated separators at a c-rate of 0.2 C and (b) corresponding discharge–charge profiles of the Li/S batteries with a TiO_2_/GO-coated separator.

The discharge–charge cyclic performance was assessed at 0.2 C in the voltage range of 1.5 to 3 V (vs Li/Li^+^). Figure 6a shows that the Li/S batteries with the TiO_2_/GO-coated separator delivered a high initial discharge capacity of 1102.8 mAh g^−1^ and a 100th cycle capacity of 843.4 mAh g^−1^, which corresponds to a capacity retention of 76.48%. On the other hand, the initial discharge capacity of Li/S batteries with a pristine and GO-coated separator were only 757.7 and 907.9 mAh g^−1^, respectively. After 100 cycles, the capacity decreased to 467.1 and 652.7 mAh g^−1^, respectively, which corresponds to a capacity retention of 61.65% and 71.89%. In addition, the TiO_2_/GO-coated separator Li/S batteries rendered a stable coulombic efficiency during charge/discharge process. The enhanced cyclic performance of the TiO_2_/GO-coated separator batteries can be attributed to the outstanding physical and chemical absorption between the TiO_2_/GO composite and the dissolved polysulfide. In addition, the TiO_2_/GO composite forms a three-dimensional structure, which can improve the active material utilization and mitigate the “shuttle effect”.

Figure 6b presents the galvanostatic discharge–charge curves of the TiO_2_/GO-coated separator batteries for the 1st, 5th, 50th and 100th cycles at a c-rate of 0.2 C. The discharge–charge curves exhibit two pairs of reduction and oxidation peaks, corresponding to the redox reactions of typical Li/S batteries. These observations are consistent with the CV curves. In addition, the plateaus in the discharge–charge profiles are almost overlapped even after the 100th cycle, indicating a stable electrochemical performance of the Li/S batteries with a TiO_2_/GO-coated separator.

**Figure 7:**
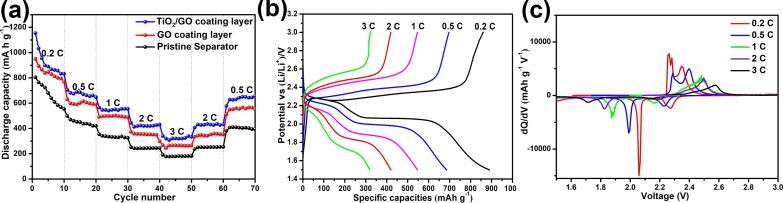
(a) Rate capability of the Li/S batteries with pristine, GO-coated and TiO_2_/GO-coated separators. (b) Charge/discharge curves and (c) corresponding d*Q*/d*V* curves of the Li/S batteries with a TiO_2_/GO-coated separator at different c-rates.

**Figure 8:**
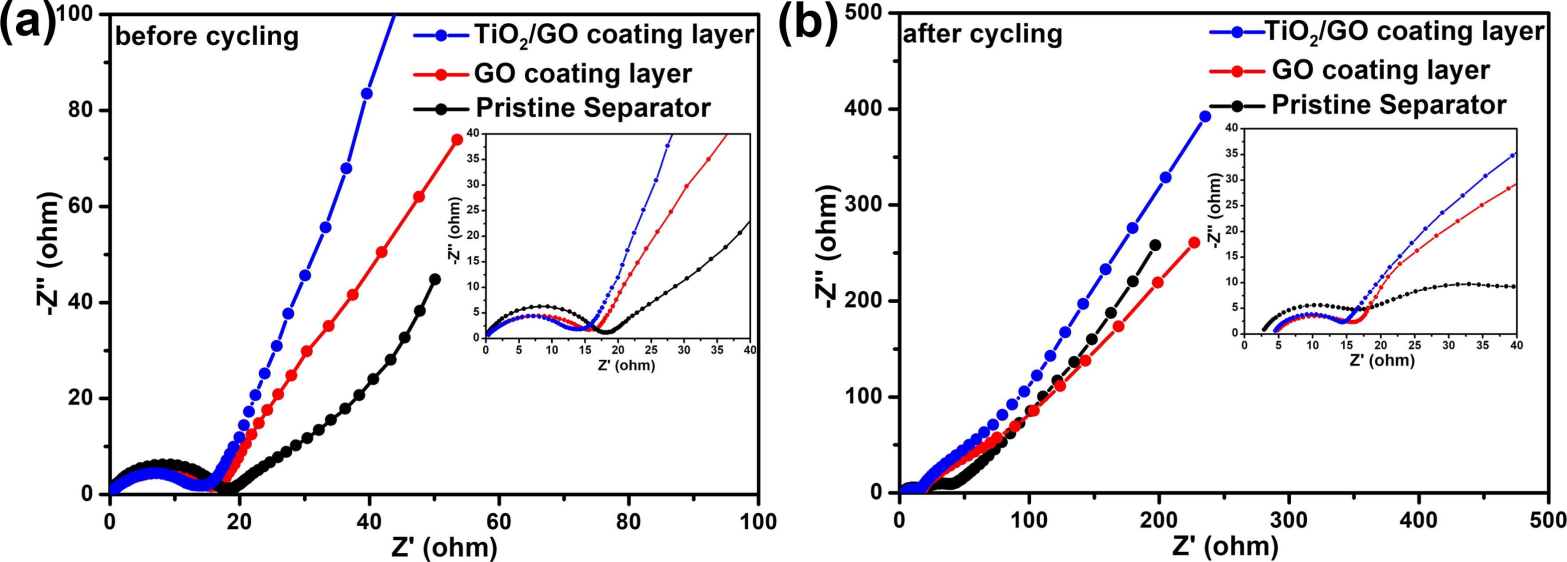
Nyquist plots of the Li/S batteries with pristine, GO-coated and TiO_2_/GO-coated separators (a) before cycling and (b) after cycling.

Figure 7a shows the rate capability of Li/S batteries with pristine, GO-coated and TiO_2_/GO-coated separators at various current rates. Over the entire discharge–charge process, the TiO_2_/GO-coated separator batteries delivered a much higher capacity than the Li/S batteries with a pristine separator. When the c-rate was increased from 0.2 to 0.5, 1 and 2 C, the TiO_2_/GO-coated separator batteries delivered a high reversible capacity of 889.7, 685.9, 546.4 and 419.7 mAh g^−1^, respectively. Even at a high c-rate of 3 C, a reasonably high reversible capacity of ≈320.8 mAh g^−1^ was delivered by the TiO_2_/GO-coated separator batteries. Moreover, once the current density was restored to a low rate (0.5 C), the TiO_2_/GO-coated separator batteries exhibited a capacity of 655 mAh g^−1^, which corresponds to a recovery of 95.5%. On the other hand, the Li/S batteries with the pristine and GO-coated separator exhibited a low capacity of ≈179.6 and 266.2 mAh g^−1^, respectively, at 3 C, which are quite lower than the Li/S batteries with the TiO_2_/GO-coated separator. For the Li/S batteries with a pristine and GO-coated separator, when the c-rate was restored to 0.5 C, only a capacity of 400.5 and 553.7 mAh g^−1^ could be restored, which indicates the poor rate capability of the Li/S batteries with a pristine separator and GO-coated separator. Figure 7b presents the discharge–charge profiles of the Li/S batteries with the TiO_2_/GO-coated separator at different current rates from 0.2 to 3 C. One should note that the shape of the voltage curves remained the same even under high current rates, such as 2 C and 3 C. Figure 7c shows the differential capacity versus voltage (d*Q*/d*V*) obtained from the discharge–charge profiles in Figure 8b. There were some shifts of the redox peaks with the increase in the current rate. However, the peak separation at a high current rate of 3 C still exhibits pronounced peaks. The excellent rate capability of the Li/S batteries with the TiO_2_/GO coated separator suggests that the migration of polysulfides has been effectively restrained due to the introduction of the separator. Moreover, the adsorption advantages of GO with oxygen functional groups and TiO_2_ with chemical bonds results in an increase in the sulfur utilization and leads to an enhanced rate stability of the Li/S batteries.

Furthermore, we have carried out electrochemical impedance spectroscopy (EIS) analysis to analyze the charge transfer kinetics in Li/S batteries with pristine and TiO_2_/GO-coated separators. Figure 8 presents the Nyquist plots of Li/S batteries with pristine and TiO_2_/GO-coated separators before and after cycling. As shown in Figure 8a, the charge transfer resistance (*R*_ct_) of the TiO_2_/GO-coated separator battery was ≈15.7 Ω, which is smaller than the Li/S battery with a pristine separator (19.2 Ω) or GO-coated separator (17.4 Ω). The lower charge transfer resistance can be ascribed to the higher conductivity of the TiO_2_/GO layer. After cycling, the *R*_ct_ of the Li/S batteries with the TiO_2_/GO-coated separator decreased to 12.6 Ω, whereas the *R*_ct_ of the Li/S batteries with the pristine and GO-coated separator reduced to 18.3 and 14.8 Ω, respectively. The lower *R*_ct_ after cycling can be ascribed to the chemical activation and redistribution of the active material [45]. In addition, an additional semicircle emerged in the EIS spectra of the Li/S batteries with the pristine separator after cycling, which suggests the dissolution of polysulfides and their deposition on the surface of the sulfur cathode. The absence of an additional semicircle in the EIS spectra of the TiO_2_/GO-coated separator batteries indicates that the presence of the TiO_2_/GO interlayer hindered the movement of polysulfides and thereby enhanced the utilization of the active material by reducing the shuttle effect.

**Figure 9:**
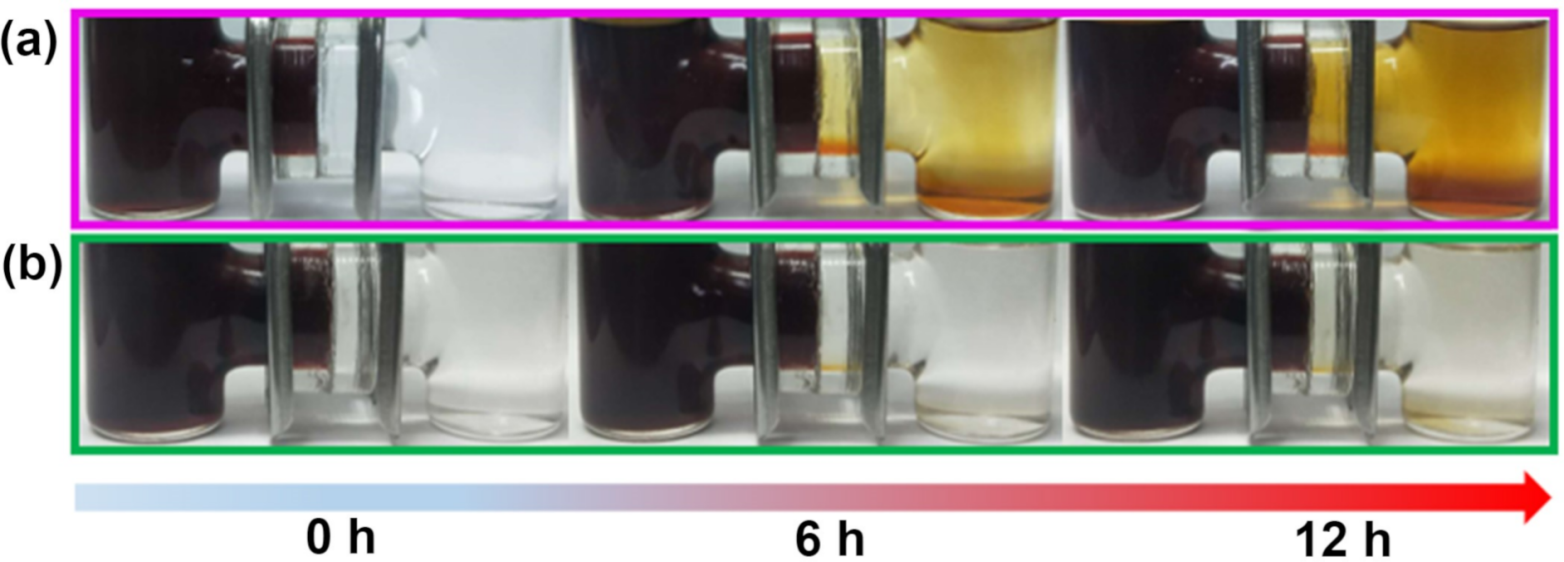
Optical images of the diffusion process of the polysulfides through the (a) pristine separator and the (b) TiO_2_/GO-coated separator.

The permeability of polysulfides through both membranes was visually analyzed by separating two compartments with either the pristine or the TiO_2_/GO-coated separator, as shown in Figure 9. The left side of the test tube was filled with anhydrous tetrahydrofuran (THF) and 1 M Li_2_S_6_ solution and the right side was filled with anhydrous THF. As shown in Figure 9a, the color of the THF solution, on the right side, changed to dark yellow with prolonged diffusion up to 12 h due to the diffusion of polysulfides from the pristine separator. On the other hand, the TiO_2_/GO-coated separator hindered the diffusion of polysulfides and exhibited a much slower color change even after 12 hours. Hence, the TiO_2_/GO-coated separator effectively adsorbed and blocked the transportation of Li_2_S_6_.

**Figure 10:**
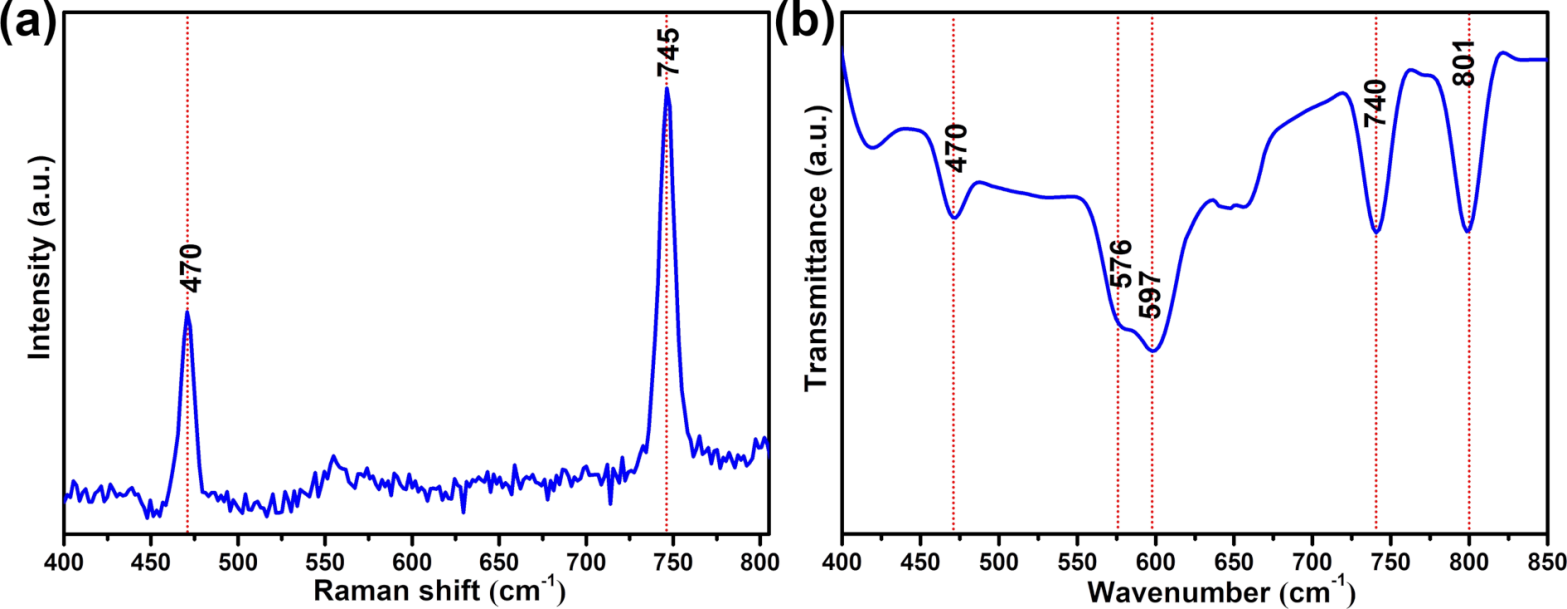
(a) Raman and (b) FTIR spectra of the Li_2_S_6_-treated TiO_2_/GO composite.

Raman and Fourier-transform infrared spectroscopy (FTIR) analysis was carried out to understand the interaction between TiO_2_/GO and polysulfides (Figure 10). The TiO_2_/GO composite was treated with a Li_2_S_6_ electrolyte (1.0 M/0.1 M LiTFSI/LiNO_3_ in DOL and DME (1:1 v/v)) via immersion for 12 h; the Li_2_S_6_-treated TiO_2_/GO material was then obtained after centrifugal separation and vacuum drying. Raman and FTIR studies of the Li_2_S_6_-treated TiO_2_/GO material clearly show the existence of an S–S stretching band at 470 cm^−1^, indicating that Li_2_S_6_ was absorbed on the surfaces of the TiO_2_/GO composite [46]. The Raman band at 745 cm^−1^ relates to a typical characteristic feature of LiTFSI in electrolyte [47]. A band that appeared at 801 cm^−1^ in the FTIR spectrum can be attributed to the S–O–C stretching, suggesting that the C of the TiO_2_/GO composite is chemically bonded to the polysulfides [48]. The FTIR spectrum of Li_2_S_6_–TiO_2_/GO revealed that the peaks at 576, 597 and 740 cm^−1^ were attributed to the asymmetric bending mode of CF_3_, the Li–O stretching and the S–N stretching of LiTFSI [49–51].

**Figure 11:**
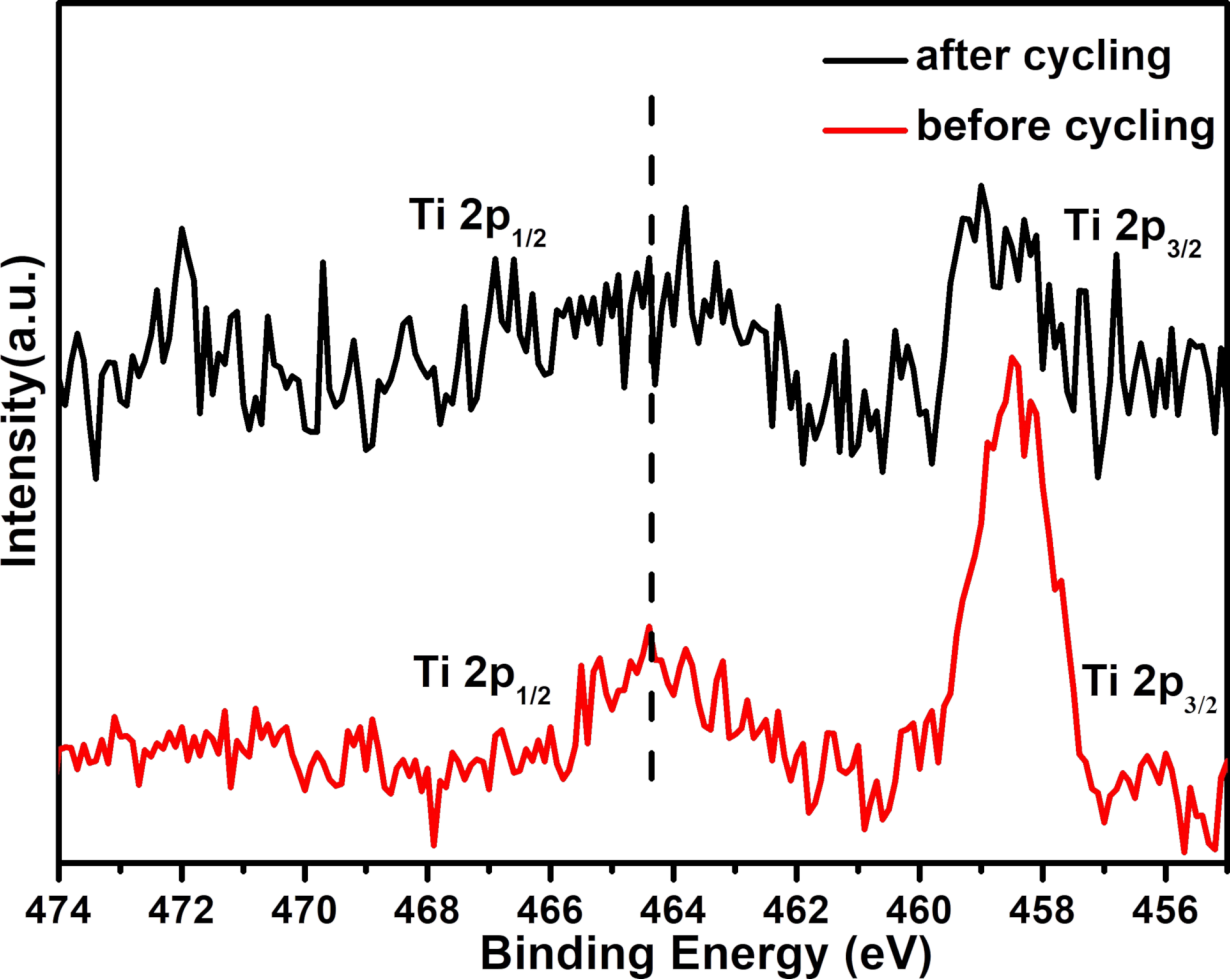
XPS spectra (Ti 2p) of the battery with a TiO_2_/GO-coated separator before and after charge/discharge cycling.

To further investigate the interaction between TiO_2_ and the polysulfides during the charge/discharge process, the XPS spectra of the Li/S batteries with the TiO_2_/GO-coated separator were recorded before and after 100 cycles, as shown in Figure 11. A broadened Ti 2p_1/2_ peak at ≈464 eV after cycling can be attributed to the presence of Ti–S interaction [32], indicating that TiO_2_ interacts with S during the charge/discharge process. The combination of TiO_2_ and sulfur effectively prevents the loss of active sulfur and improves the cyclic performance of the Li/S batteries. Based on the above results, it can be concluded that the TiO_2_/GO composite has the synergetic effects of physical and chemical interaction in inhibiting the shuttling of polysulfides. The increase of the active material utilization contributes to improvement of the cyclic performance and rate performance of Li/S batteries.

## Conclusion

In summary, a lightweight TiO_2_/GO coating, applied as an interlayer for Li/S batteries, has been prepared by using a simple method. The hierarchically porous TiO_2_ nanoparticles are tightly wrapped in GO sheets and formed a 3D network structure, which can capture the polysulfides by physical and chemical adsorption and buffer the volumetric change of the sulfur cathode during the charge/discharge process. As a result, the Li/S batteries with the TiO_2_/GO-coated separator exhibited a higher capacity, excellent rate performance and superior cyclic stability as compared to the Li/S batteries with a pristine or GO-coated separator. With the TiO_2_/GO-coated separator, the Li/S batteries still exhibited a high specific capacity of 843.4 mAh g^−1^ after 100 cycles. Additionally, the discharge capacity of ≈320.8 mAh g^−1^ can be obtained even at a high current density of 3 C. The present study demonstrates the potential of the TiO_2_/GO functional interlayer in next-generation Li/S batteries and presents a novel approach to prepare metal oxide based hybrid coatings for energy storage applications.

## Experimental

### Preparation of TiO_2_/GO composite

Ti_10_Al_90_ alloy ribbons were fabricated by refining pure Al (99.9 wt %) and Ti (99.9 wt %) in an arc furnace, followed by melt spinning under an argon-protected atmosphere. The Ti_10_Al_90_ alloy ribbons were immersed in a 2 M NaOH solution for 72 h to prepare nanoporous TiO_2_ particles at ambient temperature. The resulting powder was washed several times by using deionized (DI) water (18.2 MΩ cm) and ethyl alcohol. Then, the powder was vacuum dried (−0.08 MPa) for 8 h. To synthesize the TiO_2_/GO composites, 1 g of nanoporous TiO_2_ (≈40 nm) and 100 mL of graphene oxide (GO) solution were dispersed into 100 mL of deionized water and ultrasonically mixed for 2 h, followed by continuous stirring for 12 h to obtain a stable and uniform mixture. Then, the mixture was spray-dried at a flow rate of 5 mL min^−1^, which resulted in the TiO_2_/GO composite. The inlet temperature of the spray dryer was 200 °C.

### Fabrication of TiO_2_/GO-coated separator

The coating layer was fabricated by mixing TiO_2_/GO composites (90 wt %) and poly(vinylidene fluoride) (PVDF, 10 wt %) in ultrapure water and milling for 40 min. The as-prepared slurry was coated onto the separator and dried at 60 °C in a vacuum oven for 8 h. Then, the TiO_2_/GO-coated separator was sectioned in the form of circular discs with a diameter of 18 mm. For reference, a pure GO-modified separator was fabricated using the same process.

### Synthesis of sulfur cathode

The sulfur cathode was prepared by mixing 70 wt % of elemental sulfur, 10 wt % PVDF and 20 wt % Ketjen black in *N*-methyl-2-pyrrolidone (NMP) solvent to form a slurry, which was coated onto an aluminum foil and vacuum-dried at 60 °C for 8 h. Finally, the cathodes were cut into a round shape with a diameter of 9 mm for coin-cell fabrication.

### Material characterization

The crystalline structure of the samples was examined using XRD (Rigaku-TTRIII) with a step rate of 3° min^−1^. The morphology and microstructure were observed by SEM (JEOL JSM-7100F) and TEM (JEOL JEM-2100F) with an accelerating voltage of 15 kV and 200 kV, respectively. The Raman spectra were recorded on a Raman spectrometer (Renishaw RM 2000) by using a laser with an excitation wavelength of 632.8 nm. Thermogravimetric analysis (SDTQ600) was taken under air flow (RT to 800 °C, 10 °C min^−1^). The N_2_ adsorption/desorption tests were analyzed using Brunauer–Emmett–Teller (BET) theory on a Micromeritics ASAP 2020 device. The surface composition was analyzed by XPS (VG ESCALAB MK II USA). The binding energies of all the elements were calibrated using C 1s (284.5 eV) as a reference. The FTIR spectra of the samples were recorded on a Bruker VERTEX 80 infrared spectrometer.

### Electrochemical characterization

The 2032-type coin-cells were assembled in an argon-filled glove box (MBraun). In a half-cell configuration, Li metal served as a reference electrode, TiO_2_/GO hybrid and GO membranes as separators, 1.0 M/0.1 M LiTFSI/LiNO_3_ in DOL and DME (1:1 v/v) as an electrolyte and sulfur as a cathode. The charge–discharge measurements were carried out in the voltage range of 1.5–3 V (vs Li/Li^+^) by using a multichannel Neware battery tester. CV and EIS were carried out on an electrochemical workstation (Princeton Applied Research, PARSTAT 2273). The CV scans were collected at a scanning rate of 0.1 mV s^−1^ between the voltage range of 1.5–3 V. EIS was performed in the frequency range of 100 kHz to 0.01 Hz with an amplitude of 5 mV.
